# Evaluation of the Effects of Remifentanil and Dexmedetomidine on Renal Functions in a Rat Model of Crush Injury

**DOI:** 10.3390/medicina62102020

**Published:** 2026-10-19

**Authors:** Özer Tuç, Elvin Kesimci, Huriye Bilge Tuncer, Nuran Süngü, Sevdenur Özdüzgün Polat, Aylin Sepici Dinçel, Rabia Şemsi, Ezgi Erkılıç

**Affiliations:** 1 Department of Anesthesiology and Reanimation, Bilkent City Hospital, 06800 Ankara, Turkey; ozertuc92@gmail.com (Ö.T.); bilgetuncer@yahoo.com (H.B.T.); 2 Department of Anesthesiology and Reanimation, Başkent University Ankara Hospital, 06490 Ankara, Turkey; elvinku@yahoo.com; 3 Department of Medical Pathology, Bilkent City Hospital, 06800 Ankara, Turkey; nuransungu@gmail.com (N.S.); s.ozduzgun@hotmail.com (S.Ö.P.); 4 Department of Medical Biochemistry, Faculty of Medicine, Gazi University, 06560 Ankara, Turkey; asepicidincel@gmail.com (A.S.D.); rabiasemsi2010@gmail.com (R.Ş.)

**Keywords:** crush injury, renal function, remifentanil, dexmedetomidine, acute kidney injury, rhabdomyolysis

## Abstract

*Background and Objectives*: Crush injury frequently leads to acute kidney injury (AKI) through rhabdomyolysis, and the choice of anesthetic agent may influence renal outcomes in patients requiring surgery after such injury. This study aimed to compare the effects of remifentanil and dexmedetomidine on renal injury following crush injury using biochemical markers and histopathological examination. *Materials and Methods*: In this experimental study, 28 male Wistar-Albino rats were allocated to four groups (n = 7 each): Control, Sham (crush injury and surgery without drug), Dexmedetomidine, and Remifentanil. Bilateral gastrocnemius compression for two hours induced crush injury, followed by a one-hour intravenous drug infusion and simulated laparotomy. Blood samples were collected at 0, 2, and 6 h; animals were sacrificed at 6 h and kidney tissue was harvested. Serum urea, creatinine, neutrophil gelatinase-associated lipocalin (NGAL), kidney injury molecule-1 (KIM-1), (TIMP-2) × (IGFBP7), total antioxidant capacity (TAC), and total oxidative status (TOS) were measured, and renal histopathology was evaluated. One Sham-group animal died during the experiment; the final analysis included 27 animals. *Results*: When all time points were evaluated together, serum creatinine and NGAL levels were significantly higher in the Remifentanil group than in the Dexmedetomidine group (although creatinine was already higher in the Remifentanil group at 2 h, before drug administration), whereas KIM-1 and (TIMP-2) × (IGFBP7) did not differ significantly between groups. The correlation between TAC and TOS was weakly positive early after injury and became weakly negative by 6 h. Histopathological analysis showed more pronounced necrosis, brush-border loss, and congestion in the Remifentanil group, whereas renal tissue preservation was more evident in the Dexmedetomidine group; these mixed biochemical results do not uniformly support a renoprotective conclusion. *Conclusions*: In this experimental crush injury model, dexmedetomidine was associated with more favorable early renal biochemical and histopathological findings than remifentanil, suggesting a potential renoprotective association. However, the mixed biomarker findings, small sample size, and short observation period preclude a definitive conclusion regarding direct renal protection.

## 1. Introduction

Crush injuries resulting from natural disasters, accidents, or traumatic events represent a serious clinical condition with significant morbidity and mortality. Following prolonged compression, rhabdomyolysis may occur, leading to the release of intracellular contents and an increased risk of acute kidney injury (AKI), which is one of the most important complications observed after such injuries [1,2,3].

Patients with crush injury often require surgical intervention and intensive care management. In this context, the selection of anesthetic and sedative agents may play a role not only in maintaining hemodynamic stability but also in influencing organ function, including renal outcomes. However, the potential effects of commonly used anesthetic agents on kidney injury following crush trauma have not been fully clarified. Specifically, while both remifentanil and dexmedetomidine are routinely employed in perioperative settings for patients requiring surgery after crush trauma, their differential effects on renal injury in this specific context remain uncertain and controversial. The renal effects of dexmedetomidine have been variably reported across different injury models, and the evidence for remifentanil is similarly inconsistent, justifying the need for a dedicated experimental comparison in a crush injury model.

Remifentanil is a short-acting opioid that allows rapid titration, whereas dexmedetomidine is an alpha-2 adrenergic agonist with sedative and analgesic properties. Experimental and clinical studies have suggested that these agents may differ in their effects on inflammation, oxidative stress, and organ protection, which may be particularly relevant in conditions associated with ischemia and tissue injury. Given these divergent pharmacological profiles, we hypothesized that dexmedetomidine’s sympatholytic, anti-inflammatory, and antioxidant properties would confer greater early renoprotection than remifentanil’s opioid-receptor-mediated actions in this compression-induced crush injury model.

Early detection of kidney injury is crucial in patients at risk of AKI. In recent years, several biomarkers have been introduced to identify renal injury at an earlier stage than conventional parameters. Neutrophil gelatinase-associated lipocalin (NGAL) is one of the earliest and most sensitive serum and urinary markers of AKI, detectable within 2 h of ischemic insult. Kidney injury molecule-1 (KIM-1) is expressed on proximal tubular epithelial cells following toxic or ischemic injury and serves as a sensitive indicator of tubular damage. The product of tissue inhibitor of metalloproteinases-2 (TIMP-2) and insulin-like growth factor binding protein 7 (IGFBP7), denoted as (TIMP-2) × (IGFBP7), reflects G1 cell-cycle arrest in tubular cells under stress and has been validated as an early predictor of AKI. These biomarkers were specifically selected because they detect distinct pathophysiological pathways of tubular injury—including ischemia, oxidative stress, and cell-cycle arrest—at a stage preceding measurable decline in glomerular filtration rate, making them particularly relevant for the early post-compression time window evaluated in this study. In addition, oxidative stress has been recognized as an important contributor to renal damage, and total antioxidant capacity and total oxidative status are commonly used to evaluate the balance between oxidant and antioxidant mechanisms.

This study is designed as a bilateral gastrocnemius compression model simulating aspects of crush syndrome encountered in earthquake scenarios; it should be noted that this model represents a localized compression injury rather than a complete crush syndrome model, and the terminology “earthquake simulation” should be interpreted with appropriate caution. We aimed to investigate and compare the effects of intraoperative remifentanil and dexmedetomidine administration on renal injury following blunt compression trauma using biochemical markers and histopathological evaluation.

## 2. Materials and Methods

### 2.1. Ethical Approval and Animal Care

Ethical approval for this study was obtained from the Local Animal Ethics Committee of the University of Health Sciences, Ankara Training and Research Hospital (protocol no. 0741, dated 10.08.2023). This study has been planned in accordance with the ARRIVE Guidelines. The experimental phase of the study was conducted at the Experimental and Clinical Research Laboratories of Ankara Training and Research Hospital. Blood samples were analyzed at the Department of Medical Biochemistry, Faculty of Medicine, Gazi University, and tissue samples were evaluated at the Pathology Department of Ankara Bilkent City Hospital. All researchers involved held valid animal use certificates and had received appropriate formal training.

The study was conducted in accordance with the European Convention for the Protection of Vertebrate Animals Used for Experimental and Other Scientific Purposes (ETS 123). A total of 28 male Wistar-Albino rats (mean weight approximately 250 g) were housed under controlled environmental conditions (temperature 22–25 °C, 12 h light/dark cycle) and were fed standard rat chow and water ad libitum.

### 2.2. Experimental Groups and Crush Injury Model

On the day of the experiment, the rats were randomly assigned into four groups (n = 7 per group): Control (C), Crush Injury + Surgery Without Drug (S), Dexmedetomidine (D), and Remifentanil (R). The group previously designated as “Sham” received bilateral gastrocnemius compression and laparotomy without drug infusion, and is therefore more accurately described as a “crush injury + surgery without drug” group rather than a true sham group. A true sham group would have undergone anesthesia and surgical handling without crush injury. This distinction is acknowledged as a study limitation. Group-specific interventions are summarized in Table 1.

Before any procedures, all rats received intramuscular anesthesia with ketamine (50 mg/kg) and xylazine (5 mg/kg). They were positioned on a flat surface and secured by all four limbs to prevent movement. Venous access was established via the tail vein, and the first blood sample (baseline, 0 h) was collected in a volume of 0.5–0.8 mL. Blood was drawn into Greiner Bio-One MiniCollect^®^ Complete 0.5 mL/0.8 mL CAT Serum Sep Clot Activator tubes (Kremsmünster, Austria). The samples were centrifuged at 4000 rpm for 15 min to separate the serum from blood cells. The supernatant serum was then transferred into 1.5 mL Eppendorf tubes in aliquots of 500–1000 µL. The serum samples were stored at −80 °C until the day of analysis.

To simulate crush injury, rats in the Sham (S), Dexmedetomidine (D), and Remifentanil (R) groups underwent bilateral compression of the gastrocnemius muscles under ketamine-xylazine anesthesia using a metal clamp that applied equal pressure. This compression was maintained for a duration of two hours. Peripheral cooling and changes in coloration were monitored throughout the procedure. These clinical signs served only as indirect, real-time indicators of adequate limb compression; biochemical confirmation of rhabdomyolysis severity (e.g., serum creatine kinase, myoglobin, lactate dehydrogenase, or potassium) was not performed in this study, and this is addressed explicitly as a limitation in the Section 5. After the two-hour compression period, the clamps were removed from both gastrocnemius muscles, and second-hour blood samples were collected via tail vein access.

Following the release of compression, intravenous drug infusions were administered to the respective groups through the tail vein. The Dexmedetomidine group received Sedadomid (dexmedetomidine, Koçak Farma) at a dose of 3 µg/kg as an initial bolus followed by continuous infusion at 3 µg/kg/hour. These doses were selected based on published experimental studies in rat models demonstrating sedative and potential renoprotective effects at clinically relevant concentrations, corresponding to those used in comparable rat I/R injury studies in our prior work [4] and the literature [5,6,7]. The Remifentanil group received Opiva (remifentanil, Tüm–Ekip İlaç) at a continuous infusion rate of 2 µg/kg/min. The remifentanil dose corresponds to a clinically relevant anesthetic/analgesic range for rats, as reported in comparable experimental models [8,9]. Drug administration lasted for one hour. It should be noted that hemodynamic parameters such as heart rate, blood pressure, oxygenation, respiratory rate, and body temperature were not systematically recorded during the experiment. This is acknowledged as a limitation, as dexmedetomidine and remifentanil may influence renal perfusion indirectly through hemodynamic effects, and the absence of these measurements precludes exclusion of hemodynamically mediated differences between groups.

Simultaneously, in order to simulate a one-hour surgical procedure following crush injury, midline laparotomy was performed in the Sham, Dexmedetomidine, and Remifentanil groups. The absence of a vehicle infusion control group (i.e., a group receiving saline infusion without active drug) in the Sham group is acknowledged as a limitation, as the infusion procedure itself may have minor effects that cannot be fully excluded. The abdominal cavity was opened and subsequently closed at the end of one hour.

After the two-hour muscle compression, the one-hour simulated surgery, and the drug infusion period (i.e., three hours in total from the start of compression, corresponding to the 3rd hour from baseline), the animals were maintained under ketamine-xylazine anesthesia for an additional three hours until the sixth hour from baseline was reached, at which point blood samples were collected from the inferior vena cava. Subsequently, both kidneys were harvested. Blood was withdrawn slowly and continuously until circulatory arrest occurred, achieving complete exsanguination. Animals were sacrificed by exsanguination under deep anesthesia. Death was confirmed by the absence of spontaneous respiration and cardiac activity. Right and left kidneys were stored separately in 10% formalin for histopathological evaluation. Blood sampling was performed at three different time points: 0, 2, and 6 h. One rat in the Sham group died during the experiment.

A summary of the interventions applied to each group is provided in Table 1.

### 2.3. Biochemical Analyses

Serum samples collected at 0, 2, and 6 h were analyzed at the Department of Medical Biochemistry, Faculty of Medicine, Gazi University. All laboratory parameters reported in the Results section were measured as follows.

**Conventional renal function tests:** serum urea and creatinine (mg/dL) were measured by standard automated enzymatic/colorimetric methods on a clinical chemistry analyzer.

**Acute kidney injury biomarkers:** serum neutrophil gelatinase-associated lipocalin (NGAL), kidney injury molecule-1 (KIM-1), tissue inhibitor of metalloproteinases-2 (TIMP-2), and insulin-like growth factor binding protein 7 (IGFBP7) were measured using commercially available rat-specific enzyme-linked immunosorbent assay (ELISA) kits, according to the manufacturers’ instructions. The (TIMP-2)×(IGFBP7) value was calculated as the product of the TIMP-2 and IGFBP7 concentrations.

**Oxidative stress parameters:** total antioxidant capacity (TAC) and total oxidative status (TOS) were measured using automated colorimetric methods.

**Reference (normal) values:** in adult male Wistar rats, reported reference values are 0.50 ± 0.07 mg/dL for serum creatinine and 30.9 ± 3.4 mg/dL (fasting) to 37.0 ± 5.9 mg/dL (non-fasting) for serum urea [10]. In the Control group of the present study, which underwent neither compression, surgery nor drug administration, baseline (0 h) values were 0.76 (0.03) mg/dL for creatinine and 30 (9) mg/dL for urea (Table 2). Because no universally accepted reference intervals exist for serum NGAL, KIM-1, (TIMP-2)×(IGFBP7), TAC, and TOS in rats, the values obtained in the Control group served as the internal reference range for these parameters. 

### 2.4. Histopathological Evaluation

In our study, a total of 54 kidney specimens—right and left kidneys from 27 rats—were evaluated. The excised kidney tissues were examined both macroscopically and microscopically at the Pathology Department of Ankara Bilkent City Hospital. The tissues were fixed in 10% formalin for 24 to 48 h. After measuring the macroscopic dimensions of the tissues, samples were placed into individual cassettes in a manner that allowed visibility of the cut surfaces. These samples were then processed in the Sakura Tissue-Tek VIP 6AI fully enclosed vacuum tissue processor and embedded in paraffin using the Leica EG1150 C modular embedding system. Subsequently, 4-micrometer-thick unstained sections were obtained in duplicate from each paraffin block using the Leica RM2125RT rotary microtome. The sections were stained with Hematoxylin and Eosin (H&E) and Periodic Acid–Schiff (PAS).

Both kidneys from each rat were fixed in 10% formalin, embedded in paraffin, sectioned at 4 µm, and stained with Hematoxylin and Eosin and Periodic Acid–Schiff. All slides were evaluated independently and blindly by two pathologists. Histological assessment focused on acute tubular necrosis, brush border loss, tubular dilatation, cast formation, apoptosis, congestion, inflammation, and crystal deposition. A semi-quantitative scoring system based on the percentage of tissue involvement was used.

Apoptosis was defined as cell rounding, shrinkage, and nuclear chromatin condensation. In PAS-stained sections, brush border loss on the luminal side of the tubules was assessed. Calcium-phosphate crystal presence was scored as 0 (absent) or 1 (present). Chronic inflammation was scored as 0 (none), 1 (focal, if not filling a ×10 microscopic field), or 2 (diffuse, if filling the field). All other parameters were scored as follows:

Histopathological severity was graded semi-quantitatively as follows: 0 = absent; 1 = 0–10%; 2 = 11–25%; 3 = 26–45%; 4 = 46–75%; 5 = 76–100% tissue involvement.

### 2.5. Statistical Analysis

All statistical analyses were conducted using RStudio Version 2024.12.0+467. A comprehensive statistical analysis approach was employed to evaluate biomarker changes between experimental groups. Initially, descriptive statistics were calculated, and appropriate statistical tests such as the Wilcoxon rank-sum exact test and others were applied based on variable type. Histograms and boxplots were generated to examine variable distributions and differences in means between groups.

To visualize the data, the LOESS (Locally Estimated Scatterplot Smoothing) method was applied to assess temporal changes in biomarker levels. The statistical power analysis was based on literature-referenced sample size calculations to determine whether the chosen sample size was adequate for group comparisons [4,11,12].

However, due to the small sample size, although various analyses were feasible, insufficient statistical power was achieved for multilevel categorical comparisons of certain variables. Therefore, some variables were re-categorized to enhance interpretability and statistical robustness.

To assess differences in histopathological indicators and kidney function biomarkers between groups, variables were reclassified. Initially, multinomial logistic regression models were considered, but due to statistical instability and convergence issues, these models could not be applied. The primary reason for this was the insufficient power when analyzing four treatment groups along with variables containing four to five subcategories each. To address this limitation and improve the interpretability of results, histopathological variables were simplified into two main categories: “Mild” (0–25%) and “Severe” (26–100%). Percent-based variables were similarly reclassified. This strategy was adopted to strengthen the evaluation of inter-variable relationships and to reduce the risk of type II errors (false negatives).

To determine differences between treatment groups, one-way ANOVA tests were performed, followed by post-hoc Tukey tests for pairwise comparisons in cases of statistical significance. To examine individual variability and repeated measures, linear mixed-effects models were utilized. Interaction terms were initially included to assess the effect of time on variables but were later removed from the final model due to convergence issues.

Subgroup analyses were conducted to compare rats subjected to compression versus those not compressed, and to evaluate differences between groups that received only hypnotic agents. Correlation matrices were constructed to explore associations between kidney function biomarkers, and Pearson or Spearman correlation coefficients were calculated depending on data distribution. A *p*-value of <0.05 was considered statistically significant in all analyses. Given the large number of comparisons performed across multiple biomarkers and time points, the potential for type I error inflation is acknowledged. No formal multiple-testing correction (e.g., Benjamini–Hochberg false discovery rate) was applied; therefore, all findings should be interpreted as exploratory and hypothesis-generating rather than confirmatory. In addition, because the right and left kidneys from the same animal are not statistically independent, analyzing them as separate observations in the histopathological comparisons may introduce pseudoreplication and artificially narrow confidence intervals. This non-independence is acknowledged as a methodological limitation, and the histopathological findings should accordingly be interpreted with caution rather than as fully independent replicates.

## 3. Results

### 3.1. Biochemical Findings

Multiple comparisons of urea levels revealed no statistically significant differences between the groups. However, regarding creatinine levels, group R demonstrated significantly higher creatinine concentrations at the 2nd hour compared with group C (difference = 0.343, 95% CI: 0.091–0.596, *p* = 0.005). Of note, the 2 h sample was obtained after release of compression but before the start of drug infusion; this difference therefore reflects a pre-treatment imbalance and not an effect of the study drug. Similarly, group S showed significantly elevated creatinine levels at the 6th hour relative to group C (difference = 0.295, 95% CI: 0.031–0.558, *p* = 0.025) (Table 2).

No statistically significant differences were found between the groups in terms of (TIMP-2)(IGFBP7) and KIM-1 levels. However, when NGAL levels were compared, group S showed lower levels at the 2nd hour compared with the other groups.

When evaluating TAC, one of the indicators of the antioxidant system, group D demonstrated significantly higher TAC levels than group C at the 0th hour (difference = 0.283, 95% CI: 0.028–0.538, *p* = 0.026). At the 2nd hour, TAC levels were significantly higher in group D (difference = 0.252, 95% CI: 0.079–0.426, *p* = 0.003), group R (difference = 0.223, 95% CI: 0.050–0.396, *p* = 0.008), and group S (difference = 0.311, 95% CI: 0.138–0.484, *p* < 0.001) compared with group C. At the 6th hour, TAC levels remained significantly higher in group S compared with group C (difference = 0.493, 95% CI: 0.037–0.949, *p* = 0.031). However, the D and R groups were not statistically different from group S.

Regarding TOS, an oxidative stress marker, group S exhibited significantly higher levels at the 6th hour compared with group C (difference = 1.766, 95% CI: 0.004–3.528, *p* = 0.049). Aside from this finding, no statistically significant differences were observed at other time points or among the other groups (all *p* > 0.05).

When biomarker correlation matrices were examined, no significant correlation was observed between NGAL and KIM-1 at hour 0 (r = 0.04). At hour 2, the correlation between NGAL and KIM-1 increased markedly, reaching a moderate level (r = 0.44). By hour 6, the correlation slightly decreased (r = 0.28) but remained within the moderate range.

At hour 0, a low positive correlation was found between NGAL and (TIMP-2)(IGFBP7) (r = 0.26), whereas correlations of (TIMP-2)(IGFBP7) with NGAL (r = 0.11) and KIM-1 (r = 0.10) remained weak. At hour 6, the correlation between NGAL and (TIMP-2)(IGFBP7) increased over time, reaching its highest level (r = 0.48). In contrast, the correlation between (TIMP-2)(IGFBP7) and KIM-1 was weak and negative (r = −0.07).

In the advanced statistical evaluation, mixed regression analyses were conducted. Linear regression analyses for renal function biomarkers—creatinine and urea—were performed to assess differences between treatment groups and temporal changes.

Initially, rats were categorized into two groups: those subjected to compression and those not (i.e., Control group vs. others). Among the renal function biomarkers, only creatinine levels showed a statistically significant difference between the compressed and non-compressed groups (*p* = 0.00018), with lower creatinine levels observed in the non-compressed group. However, the main effect of time on creatinine levels was not statistically significant (*p* = 0.66).

For total antioxidant capacity, a significant difference in TAC levels was observed based on compression status (*p* = 0.000011), with significantly higher TAC levels in the compressed group (*p* < 0.00001). In contrast, time had no statistically significant effect on TAC levels (*p* = 0.96).

No statistically significant differences were found between groups for the other biomarkers: (TIMP-2)(IGFBP7) (*p* = 0.85), KIM-1 (*p* = 0.31), and NGAL (*p* = 0.46). TOS levels were significantly higher in the compressed group (*p* = 0.0038), and the effect of time on TOS levels was also statistically significant (*p* = 0.045).

No significant interaction between compression and time was detected for any of the evaluated parameters (all *p*-values > 0.05).

An analysis was conducted using linear mixed models to compare the D and R groups across all time points. The results reveal statistically significant differences between the groups in terms of creatinine and NGAL levels. Creatinine levels were significantly higher in the REMI group compared with the DEX group (*p* = 0.034), with a mean difference of 0.28 mg/dL. However, this analysis included the 2 h measurements, which were obtained before drug administration and at which creatinine was already higher in the REMI group (1.06 (0.22) vs. 0.78 (0.22) mg/dL). Between 2 and 6 h, mean creatinine decreased in the REMI group (from 1.06 to 0.90 mg/dL) and remained stable in the DEX group (from 0.78 to 0.80 mg/dL) (Table 2). The creatinine difference between the two groups therefore cannot be attributed solely to the study drugs. Similarly, NGAL levels were also significantly elevated in the REMI group (*p* = 0.049), with a mean difference of 2.09 units.

No statistically significant differences were observed between the groups regarding the other renal biomarkers. Although urea levels tended to be lower in group R, this difference did not reach statistical significance (*p* = 0.149). Likewise, (TIMP-2)(IGFBP7) levels were on average 318.2 units lower in group R compared with group D, but this difference was not statistically significant due to high intra-individual variability (*p* = 0.513). No significant difference was found between the groups in terms of KIM-1 levels either (*p* = 0.064).

As shown in Table 3, at the 6th hour, TAC levels were significantly higher in group R compared with group D (2.16 ± 0.18 vs. 1.91 ± 0.15, *p* = 0.011). Conversely, TOS levels were significantly higher in group D at the 6th hour (2.65 ± 0.77 vs. 1.52 ± 0.59, *p* = 0.011).

In the earlier time points, no significant differences were observed between the groups in terms of TOS and TAC levels.

When all groups were evaluated together and the correlation matrix between TOS and TAC parameters was examined, the relationships between oxidative stress markers at specific time points were observed. A low positive correlation was found between TOS values at 0 and 2 h (r = 0.22), while a similarly weak positive correlation was observed between TOS values at 2 and 6 h (r = 0.29).

In terms of TAC changes, the correlation between TAC at 0 and 2 h was among the highest observed (r = 0.53). The correlation between TAC values at 2 and 6 h was slightly lower but still moderate (r = 0.46).

Regarding cross-correlations between TOS and TAC, a weak positive correlation was found between TOS at 2 h and TAC at 0 h (r = 0.27), and a similarly weak positive correlation was observed between TOS at 0 h and TAC at 6 h (r = 0.28). When TOS and TAC were compared at the same time points, the correlation was weakly positive at 0 and 2 h (r = 0.24 and r = 0.12, respectively) and weakly negative at 6 h (r = –0.09).

### 3.2. Histopathological Findings

Significant differences were observed among the groups in several renal histopathological parameters, including necrosis and apoptosis, tubular dilatation, congestion, and brush border loss (*p* < 0.001) (Table 4).

When all four groups were compared, higher grades of histopathological alterations were most frequently observed in group R, in which all animals showed severe necrosis/apoptosis, brush border loss, tubular dilatation, and congestion. Group S (Crush Injury + Surgery Without Drug) also frequently showed severe brush border loss and tubular dilatation, whereas group D showed lower grades than group R in all of these parameters. In the direct comparison of the D and R groups (Table 5), the Remifentanil group showed significantly more severe necrosis/apoptosis (right and left kidneys), brush border loss (right and left kidneys), and apical blebbing (right kidney).

No statistically significant differences were found between groups regarding the presence of calcium phosphate crystals in the right and left kidneys (*p* = 0.7 and *p* > 0.9). Although the analysis of chronic inflammation and bleb formation did not reveal statistically significant differences between groups (*p*-values ranged from 0.10 to 0.3), no consistent pattern of inflammation was observed between the groups.

Regarding the distribution of calcium phosphate crystals, no distinct differences were observed between groups (*p* = 0.7068 and *p* = 1.000). For the left kidney calcium phosphate values in particular, all observations fell within the same category, resulting in a calculated *p*-value of 1.000.

According to the results of Fisher’s exact test, the distribution of histopathological indicators between the D and R groups showed significant differences in certain parameters (Table 5). Necrosis and Apoptosis (right) (*p* = 0.0046) and Necrosis and Apoptosis (left) (*p* = 0.0209): All subjects in group R (7/7) exhibited severe necrosis and apoptosis, whereas this rate was considerably lower in group D (14.3% and 28.6%, respectively).

Brush Border Loss (right) (*p* = 0.0005) and Brush Border Loss (left) (*p* = 0.0005): All animals in group D (100%) showed minimal loss of brush border, while all animals in group R exhibited severe loss.

Congestion (right and left) (*p* = 0.1923 and *p* = 0.0699): All 7 animals in group R showed severe congestion, compared with 57.1% (right) and 42.9% (left) in group D; however, these differences were not statistically significant.

Tubular Dilatation (right and left) (*p* = 0.0699 and *p* = 0.1923): No statistically significant differences were found between the two groups, although there was a tendency for greater dilatation in group R.

Casts (right and left): No significant differences were found between groups in these parameters.

Bleb formation (right and left) (*p* = 1.0000): No marked differences were observed between the two groups.

In the analysis of histopathological variables, to increase group homogeneity and enhance statistical power, mild changes between 0–25% were classified as “Mild,” and more prominent changes between 26–100% were classified as “Severe.” This categorization aimed to minimize variance issues related to low observation numbers. 

## 4. Discussion

In this experimental study evaluating the early effects of remifentanil and dexmedetomidine in rats subjected to a blunt trauma model simulating an earthquake scenario, group D was found to show more favorable findings than group R. Creatinine and NGAL levels were significantly higher in group R compared with group D when all time points were analyzed together, although the creatinine difference was already present before drug administration and therefore cannot be attributed solely to the treatment, whereas there was no statistically significant difference in urea, (TIMP-2) × (IGFBP7), and KIM-1 levels between the groups. Additionally, histopathological assessments revealed better outcomes in group D compared with group R. These findings suggest that the use of dexmedetomidine may be associated with more favorable early renal outcomes than remifentanil in this model. It should be noted, however, that the observed differences between groups may not exclusively reflect direct renoprotective effects. Dexmedetomidine and remifentanil differ substantially in their hemodynamic profiles: dexmedetomidine, as an alpha-2 adrenergic agonist, may reduce renal sympathetic tone and improve medullary perfusion, while remifentanil, as a potent opioid, may cause transient hypotension or alterations in renal blood flow. In the absence of hemodynamic monitoring data, the relative contribution of indirect hemodynamic effects versus direct renoprotective mechanisms cannot be fully determined in the current study.

Rhabdomyolysis, which can develop after mechanical trauma, may lead to serious, life-threatening complications such as acute kidney injury (AKI) [13]. Patients exposed to similar mechanical compression during earthquakes are representative of this population. The products released following rhabdomyolysis can cause AKI through several mechanisms, including intratubular obstruction due to protein precipitation in the glomeruli, renal vasoconstriction, inflammation, and tubular injury related to reactive oxygen species (ROS) production [14]. Some of these patients may require early surgical intervention in hospital settings. Therefore, early recognition and prevention of AKI, along with the use of appropriate anesthetic agents, are of critical importance.

During and after rhabdomyolysis, efforts have been made to identify early biomarkers of AKI in the bloodstream. These biomarkers allow earlier diagnosis by indicating the onset of underlying mechanisms before actual loss of kidney function occurs [15]. In the presence of toxic or ischemic injury, the production and release of these markers increase rapidly, making them detectable in blood and urine as sensitive and specific indicators [16].

Dexmedetomidine is a sedative agent that is extensively studied and widely used in both intensive care units and surgical applications. It is believed to offer significant benefits in terms of perioperative renal function [17]. However, the precise mechanism underlying this effect has not yet been fully elucidated. Sugita et al., in their study using an ischemia/reperfusion (I/R) model in rats, administered various doses of dexmedetomidine and observed significantly lower BUN and creatinine levels in the dexmedetomidine-treated groups, along with suppression of proinflammatory cytokines. The researchers concluded that even low doses of dexmedetomidine exert a protective effect [5]. Similarly, Gönüllü et al. demonstrated the renoprotective effect of dexmedetomidine in a rat renal I/R model through biochemical (BUN, creatinine, and creatinine clearance) and histopathological parameters [6]. In another study, Si et al. administered intraperitoneal dexmedetomidine in a renal I/R injury model and found reduced levels of tubular degeneration and apoptosis in the dexmedetomidine group, confirmed through both biochemical and histological analyses [7]. Zhuang et al., in their meta-analysis involving 23 studies and 2440 patients, reported that dexmedetomidine improved renal function during non-cardiac surgeries by reducing serum creatinine, BUN, cystatin C, TNF-α, and IL-6 levels [18]. Similarly, Soh et al., in a study of 108 patients undergoing aortic surgery, demonstrated that dexmedetomidine administration initiated after anesthesia induction and continued for 24 h significantly reduced the incidence of AKI [19].

However, conflicting findings also exist in the literature. Salah et al. found that dexmedetomidine infusion did not significantly alter renal function in terms of serum creatinine or creatinine clearance in patients with mild to moderate renal insufficiency undergoing cardiopulmonary bypass [20]. Likewise, Park et al. reported that intraoperative dexmedetomidine infusion did not improve renal function or provide a notable benefit in preventing delayed graft function in kidney transplant recipients, suggesting that the theoretical protective effect of dexmedetomidine against I/R injury might not translate into clinical significance in this context [21].

Remifentanil, on the other hand, is a widely used agent in clinical anesthesia. Terashi et al. suggested that anesthetic management with remifentanil may have a renoprotective effect in adult patients with chronic kidney disease undergoing orthopedic surgery [22]. In an experimental study involving 75 rats, Xiong et al. demonstrated the renoprotective effects of remifentanil against I/R injury using biochemical and histopathological assessments. By adding naloxone, they identified that this effect was mediated through opioid receptors [8]. Furthermore, Jin et al., in a rat I/R model, reported that remifentanil exerted a protective role against tubular cell apoptosis [9].

Contrarily, Sakai et al. conducted a retrospective study on patients undergoing cardiopulmonary bypass and found that remifentanil did not reduce the incidence of AKI, indicating variability in its clinical efficacy [23].

Studies simultaneously evaluating the effects of dexmedetomidine and remifentanil in rat models of I/R injury are relatively limited compared with those examining each agent individually. Erkılıç et al. investigated dexmedetomidine and remifentanil in separate groups using a rat renal I/R model. Their findings revealed that dexmedetomidine provided greater protection than remifentanil, based on both biochemical and histopathological assessments [4]. Similarly, Meinberg et al. compared the renal effects of dexmedetomidine and remifentanil in morbidly obese patients undergoing bariatric surgery and found that dexmedetomidine better preserved renal function [24].

In contrast to these previous studies, our research was based on a blunt mechanical compression injury model rather than an I/R injury mechanism. While we found no significant differences in urea levels among groups, the 2 h creatinine levels in group R were unexpectedly higher than in other groups. Since this elevation occurred prior to the administration of the intervention drug, it cannot represent a drug effect and most likely reflects individual variability among animals in their response to compression. At 6 h, creatinine levels were higher in all experimental groups than in the control group; this difference was statistically significant for group S (*p* = 0.025), whereas the D and R groups did not differ significantly from each other. However, when all time points were evaluated together using a different statistical method, creatinine levels were significantly higher in group R compared with group D. However, this combined analysis included the 2 h pre-treatment measurement, at which creatinine was already higher in group R. Moreover, mean creatinine in group R decreased between 2 and 6 h (from 1.06 to 0.90 mg/dL), whereas it remained stable in group D (from 0.78 to 0.80 mg/dL), and the two groups did not differ significantly at 6 h. The creatinine difference between the D and R groups therefore cannot be attributed solely to a treatment effect and may, at least in part, reflect pre-existing between-animal variability in the response to compression. Accordingly, the creatinine findings alone should not be interpreted as evidence of superior renoprotection by dexmedetomidine; a baseline-adjusted analysis (e.g., using the 2 h value as a covariate) in a larger sample would be required to address this question.

NGAL is widely recognized in the literature as a sensitive early biomarker for AKI in experimental models of renal I/R injury [25,26]. Studies in rat models have shown that NGAL can be easily detected in urine within 2 h after ischemia, indicating its reliability as an early marker for ischemic AKI [27]. Sabra et al. reported a reduced incidence of AKI in 30 patients who received dexmedetomidine during anesthesia for radical nephrectomy [28]. However, Bayram et al. found no significant early effect of intraoperative dexmedetomidine infusion on NGAL levels following percutaneous nephrolithotomy [29]. Similarly, Carvalho et al. reported that dexmedetomidine did not influence renal function in a rat I/R injury model. They observed no reduction in NGAL levels following dexmedetomidine administration, nor any histopathological differences in tubular injury compared with the control group [30].

In our study, we did not observe significant differences in NGAL levels between groups at the 6 h mark. At 2 h, NGAL levels were statistically similar among the D, R, and C groups, while group S showed notably lower NGAL levels. We believe this early post-compression difference may have resulted from measurement variability in biochemical assessments or sample selection fluctuations. However, in the linear regression analysis that encompassed all time points for both D and R groups, NGAL levels were significantly higher in group R.

KIM-1 is a biomarker released from proximal tubular epithelial cells during acute tubular injury, detectable in both urine and serum, and offers a valuable advantage for early diagnosis [31,32,33]. Nonetheless, its application in clinical practice remains limited. In a study by Tan et al. examining the effects of dexmedetomidine on sepsis-induced AKI in rats, KIM-1 levels were significantly lower in the dexmedetomidine group. They also reported that dexmedetomidine reduced tubular damage and inflammatory cell infiltration based on histopathological analysis [34]. In a clinical study by Sabbisetti et al., KIM-1 levels on postoperative day 2 were significantly elevated only in patients who developed AKI after cardiopulmonary bypass. The authors concluded that KIM-1 is a reliable biomarker for both acute and chronic kidney injury [35].

TIMP-2 and IGFBP7 are molecules that inhibit cell cycle progression, thus preventing abnormal proliferation of injured cells. The product of their concentrations, (TIMP-2)(IGFBP7), has emerged as a valuable early predictor of AKI [36,37,38]. Inotani et al. reported promising results using urinary (TIMP-2)(IGFBP7) and NGAL levels to predict AKI development [39]. However, we found no studies in the literature evaluating plasma (TIMP-2)(IGFBP7) concentrations.

In our study, neither (TIMP-2) × (IGFBP7) nor KIM-1 levels differed significantly between the groups at any individual time point or when all time points were evaluated together. Rather than indicating an absence of any drug effect, this null finding likely reflects the distinct pathophysiological pathways captured by these two markers—G1 cell-cycle arrest and proximal tubular epithelial injury, respectively—which may become biochemically detectable only after a longer interval than the 6 h observation window used in this study, particularly in a blunt compression model with comparatively lower acute ischemic severity than classical renal ischemia–reperfusion protocols. This apparent time-dependence is consistent with the absence of a significant between-group difference in urea, a marker whose rise is well known to lag behind creatinine and NGAL. These findings should therefore be interpreted as reflecting differing biomarker kinetics and sensitivity thresholds rather than as evidence against a renoprotective effect of either agent.

Karahan et al. investigated the effects of curcumin and intraperitoneal dexmedetomidine on renal oxidative stress and histopathological injury in a rat model of lower extremity I/R injury. In their study, the control group demonstrated marked tubular damage, basal membrane loss, and interstitial edema, whereas the treatment groups showed reduced injury. Additionally, TOS, TAC, and the oxidative stress index were assessed. The researchers found that the control group exhibited the highest TOS levels and the lowest TAC levels [40]. Yi et al., in their review, reported that remifentanil inhibits calcium overload, inflammation, oxidative stress, and apoptosis. They emphasized that remifentanil could reduce I/R injury and serve as a potential therapeutic agent for mitigating reperfusion damage [41]. Kanbak et al. implemented an infrarenal I/R model to assess lung damage related to oxidative stress and antioxidant activity. They concluded that, contrary to the antioxidant effects of propofol, remifentanil did not demonstrate protective effects. Instead, it was found to increase oxidative stress, highlighting its potentially adverse role in such contexts [42]. Bostankolu et al. explored the effects of dexmedetomidine on lower extremity tourniquet-induced I/R injury using TAC and TOS measurements. Their findings showed no additional protective effect of dexmedetomidine against tourniquet-induced I/R damage. Dexmedetomidine did not provide significant benefits in preventing lipid peroxidation or oxidative stress, suggesting its limited efficacy in such models [43].

In our study, when all groups were evaluated collectively, TOS changes showed only weak associations with TAC. The correlation between TOS and TAC at 0 and 2 h was 0.24 and 0.12, respectively, whereas at 6 h, the relationship turned negative (r = –0.09). The low correlation between these changes suggests that this balance may shift over time, and the oxidative stress response may exhibit considerable individual variability. This shift toward a weak negative correlation at 6 h may additionally reflect progressive depletion of antioxidant reserves as oxidative stress persists—a phenomenon described in prolonged tissue injury, in which continued consumption of antioxidant capacity outpaces its regeneration—rather than solely reflecting measurement variability.

Regardless of the underlying cause, trauma has been reported to induce damage to the kidneys at both the endothelial and tubular epithelial levels [44,45,46]. Koçoğlu et al. investigated the effects of intraperitoneal dexmedetomidine on histological changes caused by renal I/R injury in rats and found that the treatment group had lower histopathological scores compared with the untreated group. In the control group, they observed tubular cell swelling, cytoplasmic vacuolization, pyknotic nuclei, medullary congestion, and moderate to severe necrosis [47]. Bao et al. examined the protective effects of dexmedetomidine against I/R injury in rats by evaluating its influence on renal apoptosis, inflammatory cytokines, and TOS. Their findings demonstrated that dexmedetomidine played a protective role against I/R-induced kidney damage by inhibiting inflammation and tubular cell apoptosis, reducing TOS production, and supporting overall renal function [48].

In our study, histopathological changes were evident in all groups subjected to compression, whereas the Control group showed predominantly mild changes. In the four-group comparison, the most severe alterations were observed in group R, in which all animals showed severe (26–100%) necrosis/apoptosis, brush border loss, tubular dilatation, and congestion. Group S also showed frequent severe brush border loss and tubular dilatation, while group D showed fewer severe changes than group R in all of these parameters. When comparing only the effects of the two drugs, dexmedetomidine was associated with significantly better preservation of renal tissue in terms of necrosis/apoptosis, brush border loss, and apical blebbing, whereas the differences in tubular dilatation and congestion did not reach statistical significance.

## 5. Conclusions

Blunt trauma to the muscles, often seen following natural disasters, and the subsequent need for surgical intervention in affected individuals remain a persistent clinical reality. In this study, we simulated an earthquake-related trauma model by applying blunt compression to the lower extremity and compared the effects of dexmedetomidine and remifentanil. Our findings indicated that dexmedetomidine was associated with more favorable early renal biochemical and histopathological findings compared with remifentanil. These results suggest a potential renoprotective association of dexmedetomidine in this specific model; however, given the mixed biomarker findings (including the absence of significant differences in KIM-1 and TIMP-2 × IGFBP7 levels), the small sample size, and the short observation period, definitive conclusions regarding direct renal protection cannot be drawn. The absence of conventional rhabdomyolysis markers (creatine kinase, myoglobin, LDH, potassium), urinary biomarkers, and hemodynamic monitoring data represents important limitations that should be addressed in future studies. However, considering that both agents are widely used in anesthetic practice, we believe that further comparative studies are still warranted to better elucidate their respective effects. The present study has several important limitations that should be explicitly acknowledged. First, the small sample size (n = 7 per group, with n = 6 in the Sham group due to one animal death) limits statistical power. Second, the observation period was restricted to six hours from baseline, precluding evaluation of long-term renal functional outcomes. Third, conventional markers of rhabdomyolysis—including serum creatine kinase, myoglobin, potassium, and lactate dehydrogenase—were not measured, making it difficult to fully confirm the severity and validity of the crush injury model; this should be addressed in future studies. Fourth, urinary biomarkers (urine NGAL, urine KIM-1, urine TIMP-2 × IGFBP7) and urine output were not evaluated. Fifth, hemodynamic parameters were not monitored, precluding exclusion of indirectly mediated renal effects. Sixth, no vehicle infusion control group was included. Seventh, both kidneys from each animal were analyzed as separate observations; since they are biologically paired, inter-kidney correlations should be accounted for in future analyses. Eighth, this study used only male rats, and sex-related differences in renal responses to crush injury or anesthetic agents cannot be excluded. Ninth, all groups requiring intervention—including the Dexmedetomidine and Remifentanil groups—received background ketamine-xylazine anesthesia for the compression procedure and simulated surgery; although this exposure was applied uniformly across groups, ketamine-xylazine itself has documented hemodynamic and renal effects that cannot be entirely excluded as a confounding factor influencing the observed between-group differences. Tenth, serum creatinine was already higher in the Remifentanil group at 2 h, i.e., before drug administration; this pre-treatment imbalance may have contributed to the creatinine difference observed between the Dexmedetomidine and Remifentanil groups.

## Figures and Tables

**Table 1:** Group-specific interventions.

Group	Control (C)	Sham (S)	Dexmedetomidine (D)	Remifentanil (R)
n	7	7	7	7
Bilateral gastrocnemius compression	No	Yes	Yes	Yes
Abdominal incision/closure	No	Yes	Yes	Yes
Drug administration	No	No	Yes	Yes
Blood sampling (0/2/6 h)	Yes	Yes	Yes	Yes
Bilateral renal tissue collection	Yes	Yes	Yes	Yes

**Table 2:** Temporal changes in renal function biomarkers across groups (n = 27). Values are mean (standard deviation); *p*-values from ANOVA. * *p* < 0.05.

Biomarker	C	D	R	S	*p*-Value (ANOVA)
Urea (0 h)	30 (9)	27 (15)	31 (13)	33 (20)	0.9
Urea (2 h)	33 (17)	34 (10)	20 (6)	31 (13)	0.12
Urea (6 h)	23 (14)	31 (13)	24 (11)	36 (17)	0.3
Creatinine (0 h)	0.76 (0.03)	0.81 (0.06)	0.80 (0.05)	0.82 (0.05)	0.079
Creatinine (2 h)	0.72 (0.18)	0.78 (0.22)	1.06 (0.22)	0.87 (0.10)	0.011 *
Creatinine (6 h)	0.65 (0.09)	0.80 (0.13)	0.90 (0.09)	0.95 (0.31)	0.008 *

**Table 3:** Temporal changes in antioxidant/oxidative-stress parameters in the Dexmedetomidine and Remifentanil groups. Values are mean (standard deviation); ^1^ Wilcoxon rank-sum exact test. * *p* < 0.05.

Antioxidant Parameter	Dexmedetomidine (n = 7)	Remifentanil (n = 7)	*p*-Value ^1^
TAC (0 h)	2.18 (0.17)	2.12 (0.15)	0.6
TAC (2 h)	1.99 (0.10)	1.96 (0.14)	>0.9
TAC (6 h)	1.91 (0.15)	2.16 (0.18)	0.011 *
TOS (0 h)	1.71 (1.30)	1.59 (0.74)	>0.9
TOS (2 h)	3.12 (1.76)	2.37 (0.99)	0.5
TOS (6 h)	2.65 (0.77)	1.52 (0.59)	0.011 *

**Table 4:** Histopathological changes among the four experimental groups. Values are n (%); percentages are within-group (column) percentages, calculated with the number of animals in each group as the denominator (C, D, and R: n = 7; S: n = 6, owing to one animal death); ^1^ Fisher’s exact test. * *p* < 0.05.

Pathological Finding	C (n = 7)	D (n = 7)	R (n = 7)	S (n = 6)	*p*-Value ^1^
Necrosis/Apoptosis (Right)					<0.001 *
Mild (0–25%)	7 (100%)	6 (85.7%)	0 (0%)	5 (83.3%)	
Severe (26–100%)	0 (0%)	1 (14.3%)	7 (100%)	1 (16.7%)	
Necrosis/Apoptosis (Left)					<0.001 *
Mild (0–25%)	7 (100%)	5 (71.4%)	0 (0%)	4 (66.7%)	
Severe (26–100%)	0 (0%)	2 (28.6%)	7 (100%)	2 (33.3%)	
Brush Border Structures (Right)					<0.001 *
Mild (0–25%)	7 (100%)	7 (100%)	0 (0%)	1 (16.7%)	
Severe (26–100%)	0 (0%)	0 (0%)	7 (100%)	5 (83.3%)	
Brush Border Structures (Left)					<0.001 *
Mild (0–25%)	7 (100%)	7 (100%)	0 (0%)	1 (16.7%)	
Severe (26–100%)	0 (0%)	0 (0%)	7 (100%)	5 (83.3%)	
Tubular Dilatation (Right)					<0.001 *
Mild (0–25%)	7 (100%)	4 (57.1%)	0 (0%)	1 (16.7%)	
Severe (26–100%)	0 (0%)	3 (42.9%)	7 (100%)	5 (83.3%)	
Tubular Dilatation (Left)					<0.001 *
Mild (0–25%)	7 (100%)	3 (42.9%)	0 (0%)	0 (0%)	
Severe (26–100%)	0 (0%)	4 (57.1%)	7 (100%)	6 (100%)	
Congestion (Right)					<0.001 *
Mild (0–25%)	7 (100%)	3 (42.9%)	0 (0%)	3 (50.0%)	
Severe (26–100%)	0 (0%)	4 (57.1%)	7 (100%)	3 (50.0%)	
Congestion (Left)					0.012 *
Mild (0–25%)	6 (85.7%)	4 (57.1%)	0 (0%)	3 (50.0%)	
Severe (26–100%)	1 (14.3%)	3 (42.9%)	7 (100%)	3 (50.0%)	
Apical Blebbing (Right)					0.002 *
Mild (0–25%)	7 (100%)	7 (100%)	2 (28.6%)	5 (83.3%)	
Severe (26–100%)	0 (0%)	0 (0%)	5 (71.4%)	1 (16.7%)	
Apical Blebbing (Left)					0.10
Mild (0–25%)	7 (100%)	5 (71.4%)	3 (42.9%)	5 (83.3%)	
Severe (26–100%)	0 (0%)	2 (28.6%)	4 (57.1%)	1 (16.7%)	
Chronic Inflammation (Right)					0.3
Absent	2 (28.6%)	0 (0%)	0 (0%)	1 (16.7%)	
Focal	1 (14.3%)	3 (42.9%)	5 (71.4%)	3 (50.0%)	
Diffuse	4 (57.1%)	4 (57.1%)	2 (28.6%)	2 (33.3%)	
Chronic Inflammation (Left)					0.2
Absent	2 (28.6%)	1 (14.3%)	0 (0%)	0 (0%)	
Focal	3 (42.9%)	2 (28.6%)	6 (85.7%)	5 (83.3%)	
Diffuse	2 (28.6%)	4 (57.1%)	1 (14.3%)	1 (16.7%)	
Calcium Phosphate Crystals (Right)					0.7068
Absent	7 (100%)	6 (85.7%)	5 (71.4%)	5 (83.3%)	
Present	0 (0%)	1 (14.3%)	2 (28.6%)	1 (16.7%)	
Calcium Phosphate Crystals (Left)					>0.9
Absent	7 (100%)	7 (100%)	6 (85.7%)	6 (100%)	
Present	0 (0%)	0 (0%)	1 (14.3%)	0 (0%)	

**Table 5:** Histopathological parameters compared directly between the Dexmedetomidine (D) and Remifentanil (R) groups. Values are n (%); percentages are within-group (column) percentages (n = 7 per group); ^1^ Fisher’s exact test. * *p* < 0.05.

Histopathological Parameter	D (n = 7)	R (n = 7)	*p*-Value ^1^
Necrosis/Apoptosis (Right)			0.005 *
Mild (0–25%)	6 (85.7%)	0 (0%)	
Severe (26–100%)	1 (14.3%)	7 (100%)	
Necrosis/Apoptosis (Left)			0.021 *
Mild (0–25%)	5 (71.4%)	0 (0%)	
Severe (26–100%)	2 (28.6%)	7 (100%)	
Brush Border Structures (Right)			<0.001 *
Mild (0–25%)	7 (100%)	0 (0%)	
Severe (26–100%)	0 (0%)	7 (100%)	
Brush Border Structures (Left)			<0.001 *
Mild (0–25%)	7 (100%)	0 (0%)	
Severe (26–100%)	0 (0%)	7 (100%)	
Tubular Dilatation (Right)			0.070
Mild (0–25%)	4 (57.1%)	0 (0%)	
Severe (26–100%)	3 (42.9%)	7 (100%)	
Tubular Dilatation (Left)			0.2
Mild (0–25%)	3 (42.9%)	0 (0%)	
Severe (26–100%)	4 (57.1%)	7 (100%)	
Congestion (Right)			0.2
Mild (0–25%)	3 (42.9%)	0 (0%)	
Severe (26–100%)	4 (57.1%)	7 (100%)	
Congestion (Left)			0.070
Mild (0–25%)	4 (57.1%)	0 (0%)	
Severe (26–100%)	3 (42.9%)	7 (100%)	
Apical Blebbing (Right)			0.021 *
Mild (0–25%)	7 (100%)	2 (28.6%)	
Severe (26–100%)	0 (0%)	5 (71.4%)	
Apical Blebbing (Left)			0.6
Mild (0–25%)	5 (71.4%)	3 (42.9%)	
Severe (26–100%)	2 (28.6%)	4 (57.1%)	
Calcium Phosphate Crystals (Right)			>0.9
Absent	6 (85.7%)	5 (71.4%)	
Present	1 (14.3%)	2 (28.6%)	
Calcium Phosphate Crystals (Left)			>0.9
Absent	7 (100%)	6 (85.7%)	
Present	0 (0%)	1 (14.3%)	

## Data Availability

The data presented in this study are available on request from the corresponding author due to (specify the reason for the restriction).
